# Using mHealth to Increase the Reach of Local Guidance to Health Professionals as Part of an Institutional Response Plan to the COVID-19 Outbreak: Usage Analysis Study

**DOI:** 10.2196/20025

**Published:** 2020-08-19

**Authors:** Olivier Windisch, Ido Zamberg, Marie-Céline Zanella, Angèle Gayet-Ageron, Katherine Blondon, Eduardo Schiffer, Thomas Agoritsas

**Affiliations:** 1 Division of Urology Department of Surgery University Hospitals of Geneva Geneva Switzerland; 2 Division of General Internal Medicine Department of Medicine University Hospitals of Geneva Geneva Switzerland; 3 Faculty of Medicine University of Geneva Geneva Switzerland; 4 School of Education Johns Hopkins University Baltimore, MD United States; 5 Division of Infectious Diseases Department of Medicine University Hospitals of Geneva Geneva Switzerland; 6 Division of Clinical Epidemiology Department of Community Health and Medicine University Hospitals of Geneva Geneva Switzerland; 7 Medical Directorate University Hospitals of Geneva Geneva Switzerland; 8 Division of Anesthesiology Department of Anesthesiology, Clinical Pharmacology, Intensive Care and Emergency Medicine University Hospitals of Geneva Geneva Switzerland; 9 Department of Health Research Methods, Evidence, and Impact McMaster University Hamilton, ON Canada

**Keywords:** COVID-19, smartphone, mHealth, information dissemination, health professionals, health administration, health apps

## Abstract

**Background:**

The ongoing coronavirus disease (COVID-19) pandemic forced health jurisdictions worldwide to significantly restructure and reorganize their medical activities. In response to the rapidly evolving body of evidence, a solid communication strategy is needed to increase the reach of and adherence to locally drafted and validated guidance to aide medical staff with COVID-19–related clinical decisions.

**Objective:**

We present a usage analysis of a dedicated mobile health (mHealth) platform as part of an institutional knowledge dissemination strategy of COVID-19–related guidance to all health care workers (HCWs) in a large academic hospital.

**Methods:**

A multidisciplinary team of experts drafted local guidance related to COVID-19. In total, 60 documents and 17 external links were made available through the platform. Documents were disseminated using a recently deployed mHealth platform for HCWs. Targeted dissemination of COVID-19–related content began on March 22, 2020. Using a third-party statistics tool, data concerning user activity and content use was anonymously collected. A quantitative analysis of user activity was performed over a 4-month period, separated into 3 periods: 2 months before (Period A), 2 weeks after (Period B), and 6 weeks following (Period C) targeted dissemination. Regional epidemiological data (daily new COVID-19 cases and total COVID-19–related hospitalizations) was extracted from an official registry.

**Results:**

During the study period, the platform was downloaded by 1233 new users. Consequently, the total number of users increased from 1766 users before Period A to a total of 2999 users at the end of Period C. We observed 27,046 document views, of which 12,728 (47.1%) were COVID-19–related. The highest increase in activity occurred in Period B, rapidly following targeted dissemination, with 7740 COVID-19–related content views, representing 71.2% of total content views within the abovementioned period and 550 daily views of COVID-19–related documents. Total documents consulted per day increased from 117 (IQR 74-160) to 657 (IQR 481-1051), *P*<.001. This increase in activity followed the epidemiological curbing of newly diagnosed COVID-19 cases, which peaked during Period B. Total active devices doubled from 684 to 1400, daily user activity increased fourfold, and the number of active devices rose from 53 (IQR 40-70) to 210 (IQR 167-297), *P*<.001. In addition, the number of sessions per day rose from 166 (IQR 110-246) to 704 (IQR 517-1028), *P*<.001. A persistent but reduced increase in total documents consulted per day (172 [IQR 131-251] versus 117 [IQR 74-160], *P*<.001) and active devices (71 [IQR 64-89] versus 53 [IQR 40-70]) was observed in Period C compared to Period A, while only 29.8% of the content accessed was COVID-19–related. After targeted dissemination, an immediate increase in activity was observed after push notifications were sent to users.

**Conclusions:**

The use of an mHealth solution to disseminate time-sensitive medical knowledge seemed to be an effective solution to increase the reach of validated content to a targeted audience.

## Introduction

Since the World Health Organization (WHO) declared the novel coronavirus (severe acute respiratory syndrome coronavirus 2 [SARS-CoV-2]) a pandemic on March 11, 2020, it has spread rapidly worldwide. As of July 26, 2020, there were more than 15.7 million confirmed cases and more than 640,000 COVID-19–related deaths [1,2]. Authorities and hospitals worldwide have braced themselves to face the unprecedented challenge of providing adequate patient care while trying to avoid health care system saturation and guaranteeing maximum protection for health care workers (HCWs) from nosocomial infections and psychological consequences [3,4].

To respond to the present challenge, health institutions worldwide have been forced to deeply reorganize and reprioritize their daily activities. Hospitals have been restructured to better streamline the increasing number of patients and to separate suspected and confirmed COVID-19 cases from patients without COVID-19 to avoid nosocomial infections [5]. In certain countries, field hospitals and drive-through screening facilities have been built to reduce the load on local health institutions and decrease the community transmission rate [6,7]. Medical and surgical units have been converted for the care of patients with COVID-19 and intensive care units as well as operating rooms were reshaped to prepare for a large number of patients in need of acute respiratory support [8]. Medical staff have also been delocalized or newly recruited to help in such units, with very little initial training on the management of COVID-19. In parallel, local, national, and international health institutions have dealt with important scientific knowledge gaps concerning SARS-CoV-2 infection vectors, transmission dynamics, epidemiology, and treatment possibilities [9].

At the University Hospitals of Geneva (HUG), all nonemergency surgeries were cancelled to mobilize anesthesiology staff to the intensive care unit and to spare essential medical equipment. Outpatient clinic activity was reduced significantly and appointments were conducted via telephone and telemedicine consultations when feasible. Non–COVID-19 medical and surgical activities were transferred to other local hospitals, and COVID-19–related activity was centralized at the authors’ institution. New dedicated wards were created, and others were transformed to admit patients with SARS-CoV-2 under the responsibility of the General Internal Medicine Division. More than 120 medical and surgical staff from a large number of departments (including gynecology and pediatrics) were recruited to the abovementioned units, as well as to the Intensive Care and Emergency Departments to respond to the increasing number of COVID-19–related hospitalizations and consultations. In addition, military paramedical personnel and advanced medical students were recruited to support patient care.

To respond to the rapidly evolving body of evidence and staff training needs, a local multidisciplinary team of experts was formed, with the mission to critically appraise and adopt published evidence and produce institutional guidance adapted to local epidemiology and resources. As new and sometimes contradictory COVID-19–related evidence was published on a daily basis, the guidance was updated frequently as new information became available. Thus, there was a crucial need to be able to disseminate the drafted guidance to medical staff and to rapidly communicate any updates.

Communication between health institutions and health professionals is critical, and many steps exist in the transmission of information from health authorities and local medical leadership to health care providers. Previous research suggests there is a discrepancy between the perceived ability of health authorities or medical leadership to disseminate information and the actual delivery of information to the targeted audience [10,11]. A solid communication strategy is therefore crucial, especially in a pandemic context, where information is time-sensitive, and evidence is constantly and rapidly evolving [12]. Recruiting medical staff from different clinical backgrounds and medical students, who are possibly unfamiliar with the equipment and specific procedures used in COVID-19 care, means that more supervision and training is required, further underscoring the need for readily available validated information. Mobile health technology solutions, referred to as mHealth, are increasingly used and gaining relevance among health professionals, and may represent an interesting solution to disseminate information to health care providers [13,14]. Such mHealth solutions are not only used to disseminate information to HCWs, but also to patients or guardians. One example is ICNexchange, which is a Pinterest-based platform that provides medical information to pediatric patients under treatment for inflammatory bowel disease. ICNexchange’s feasibility, acceptability, and utility was shown in a recent quantitative analysis of user activity [15].

We present here an assessment of an mHealth platform used to disseminate institutional COVID-19–related guidance to medical staff in our institution during the pandemic as part of hospital’s reorganization and knowledge dissemination strategy. Our hypothesis was that the use of such an mHealth solution would increase the reach of validated knowledge to HCWs. Since adequate reach to HCWs is difficult to assess, we performed a quantitative assessment using real-time and anonymous statistics to assess user activity and content use before, shortly after, and over an extended period following the targeted dissemination of information on the platform.

## Methods

### mHealth Solution Description

Our team developed a mobile smartphone platform called HeadToToe to help medical staff easily access locally validated and endorsed medical content [16,17]. It provides an institutional knowledge dissemination solution and has been organized by medical specialty to offer internationally and locally validated and up-to-date medical guidance in the form of documents, videos, and clinical scores, previously selected and validated by senior physicians. The platform offers an administration interface allowing easy content updating; updates are promptly and automatically available for users. All content has a planned obsolescence (ie, it will expire after a predefined date) set by the content’s owner, guaranteeing that all available content on the platform is up-to-date. In addition, automatic and anonymous real-time statistics are collected with Yahoo Flurry [18], which provides data concerning user activity and content viewing patterns. A detailed description of the instrument’s development process and features is described in previous publications [16,17]. The platform is available on iOS and Android and both versions provide the same features and user experience to HCWs.

### Guidelines Team Activity

Due to the urgency of the current situation and abovementioned scientific knowledge gaps, and as part of the institutional response plan to COVID-19, a multidisciplinary team of experts was created, which was independent from the mHealth platform team. The institutional team’s mission was that of critically appraising the available information on COVID-19 and creating trustworthy, actionable, and evidence-based local guidance for the hospital’s HCWs considering local epidemiology and resources. Topics covered were local containment measures, HCW protection procedures, diagnostic criteria, testing strategies, patient orientation and triage, inpatient management and treatment strategies, outpatient follow-up and management, pharmacological considerations and interactions, and specific information for patients. As new studies and information were published on a daily basis, guidance was frequently modified and updated in accordance with recent evidence.

The objective of the team was to disseminate information to all of the hospital’s HCWs to ensure, to the best of their abilities, HCWs were adequately informed and there were no unwarranted differences in patient care.

### COVID-19 Guidance Dissemination

COVID-19–related content was progressively made available through a HUG website and the mHealth platform. After a successful 18-day pilot implementation in the Children’s Hospital of the HUG [12], institutional leadership decided to deploy the platform to all medical departments on March 22, 2020.

At the time of analysis, a total of 60 local procedures were drafted and made available through the platform with an additional 17 links to governmental websites and other validated resources.

The guidance topics distribution was as follows: 4 procedures concerning general overview and identification of suspected cases, 3 concerning orientation strategies, 13 concerning inpatient management, 21 procedures related to pharmacological considerations, 10 for acute care management, 6 for outpatient management, and 3 procedures related to COVID-19–related death.

### Data Analysis and Statistical Methods

User activity and content viewing patterns were compared between three distinct periods. Period A was defined as the 2-month period preceding the targeted dissemination of COVID-19–related content in the institution (from January 21, 2020, to March 21, 2020). Period B was defined as the 14-day period beginning on the day of targeted dissemination, in parallel with the hospital reorganization and reallocation of medical staff (from March 22, 2020, to April 4, 2020). Period C was an extended period of 6 additional weeks after targeted dissemination (from April 5, 2020, to May 21, 2020). Since our mHealth solution was already used during Period A in the Children’s Hospital, pediatric documents regarding COVID-19 were already available on the platform at the time of targeted dissemination. Data retrieved from Yahoo Flurry included the number of active devices, new device installations (defined further as new devices), and total sessions, as well as data concerning content use. To assess the use of COVID-19–related documents, we collected and classified all documents used during the three periods, and manually identified which were COVID-19–related. Local epidemiologic data was extracted from an official and public national registry of the number of new daily cases of COVID-19 and total hospitalizations in the canton of Geneva, Switzerland [19]. It should be noted that all regional confirmed cases of COVID-19 requiring hospitalization were centralized in our institution (HUG). Epidemiological data were graphically presented and superimposed on the platform’s activity. Data are presented by their mean, median, and interquartile range (IQR) for continuous variables; relative frequencies and percentages are used for categorical variables. We compared data between the time periods using a nonparametric Mann-Whitney *U* test. Stata (Version 16, StataCorp LLC) was used for all statistical analysis [20]. Significance refers to a *P* value <.05.

## Results

By the end of the study, 1236 new users downloaded the app. The total number of users increased from 1766 users before Period A to 2999 users at the end of Period C. In total, documents were consulted 27,046 times during the study period, of which 12,728 (47.1%) were COVID-19–related.

**Figure 1:**
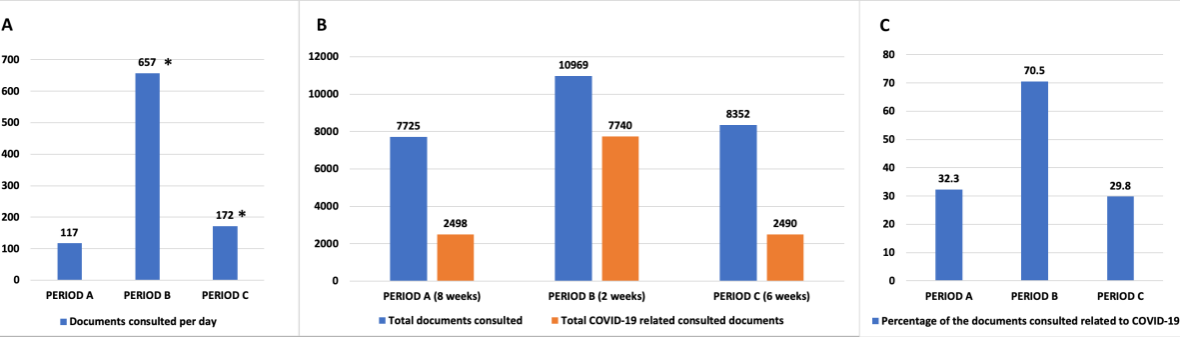
Total and coronavirus disease–related document views distribution within all three observation periods. An asterisk indicates a significant increase when compared to Period A (*P*<.01). COVID-19: coronavirus disease.

During Period B, shortly following targeted dissemination, the mobile app was downloaded 912 times and the total active devices using the platform doubled. User activity increased significantly, observed by a four-fold increase in the number of active devices per day (53 versus 211, *P*<.001) and the number of sessions per day (166 versus 704, *P*<.001). The average time spent on the app per device per day increased by 24% (*P*=.02). Table 1 provides additional information on app use. Figure 1 shows a significant increase in document views per day (117 versus 657, *P*<.001), of which 70.5% were COVID-19–related.

**Table 1:** General data concerning app use^a^.

App use variables	Period A, median (IQR)	Period B, median (IQR)	Change (B vs A), %	*P* value (B vs A)	Period C, median (IQR)	Change (C vs A), %	*P* value (C vs A)
Daily document views	114 (74-160)	657 (481-1051)	+671	<.001	172	+51	<.001
Active devices per day	53 (40-70)	211 (167-297)	+296	<.001	71 (64-89)	+34	<.001
Number of sessions per day	166 (110-246)	704 (517-1028)	+322	<.001	225 (185-282)	+36	<.001
New devices per day	2 (1-5)	26 (13-56)	+1200	<.001	2 (1-4)	0	.75
Time per device per day (minutes)	4.0 (3.1-5.1)	5.0 (4.5-5.6)	+24	.02	3.9 (3.3-4.7)	–3	.88

^a^Period A is defined as the 2-month period preceding targeted dissemination (from January 21, 2020, to March 21, 2020). Period B is defined as a 14-day period beginning on the day of targeted dissemination (from March 22, 2020 to April 4, 2020). Period C is defined as a 6-week period (from April 5, 2020 to May 21, 2020) following targeted dissemination. Where the *P* value was <.05 in a two-sided test, significance was considered reached.

In Period C, there were an additional 136 downloads of the mHealth platform. User activity had a persistent increase in Period C compared to Period A, as measured by total active devices during the period (1029 in Period C versus 684 in Period A), with a 35% increase in daily active devices (71 in Period C versus 53 in Period A, *P*<.001), and number of sessions per day (225 versus 166, *P*<.001). Figure 1 shows a persistent increase in documents consulted per day compared to Period A (172 in Period C versus 117 in Period A, *P*<.001), while only 29.8% of the total documents consulted were COVID-19–related. Global user activity was reduced compared to Period B as shown in Table 1. Table 2 provides additional details concerning new and active devices and reveals that a higher proportion of users used the iOS app throughout all periods.

**Table 2:** New and active devices and associated operating systems.

Device details		Period A (before dissemination), n (%)		Period B (Weeks 1-2 since dissemination), n (%)		Change, %		Period C (Weeks 3-8 after dissemination), n (%)		Change, %	
**Active devices**											
	Total		684 (100)		1400 (100)		+105		1029 (100)		+50
	iOS		464 (67.8)		892 (63.7)		+92		705 (68.5)		+52
	Android		220 (32.2)		508 (36.3)		+131		324 (31.5)		+47
**New devices**											
	Total		185 (100)		912 (100)		+392		136 (100)		–32
	iOS		121 (65.4)		597 (65.5)		+393		90 (66.2)		–26
	Android		64 (34.6)		313 (34.5)		+389		46 (33.8)		–28

**Figure 2:**
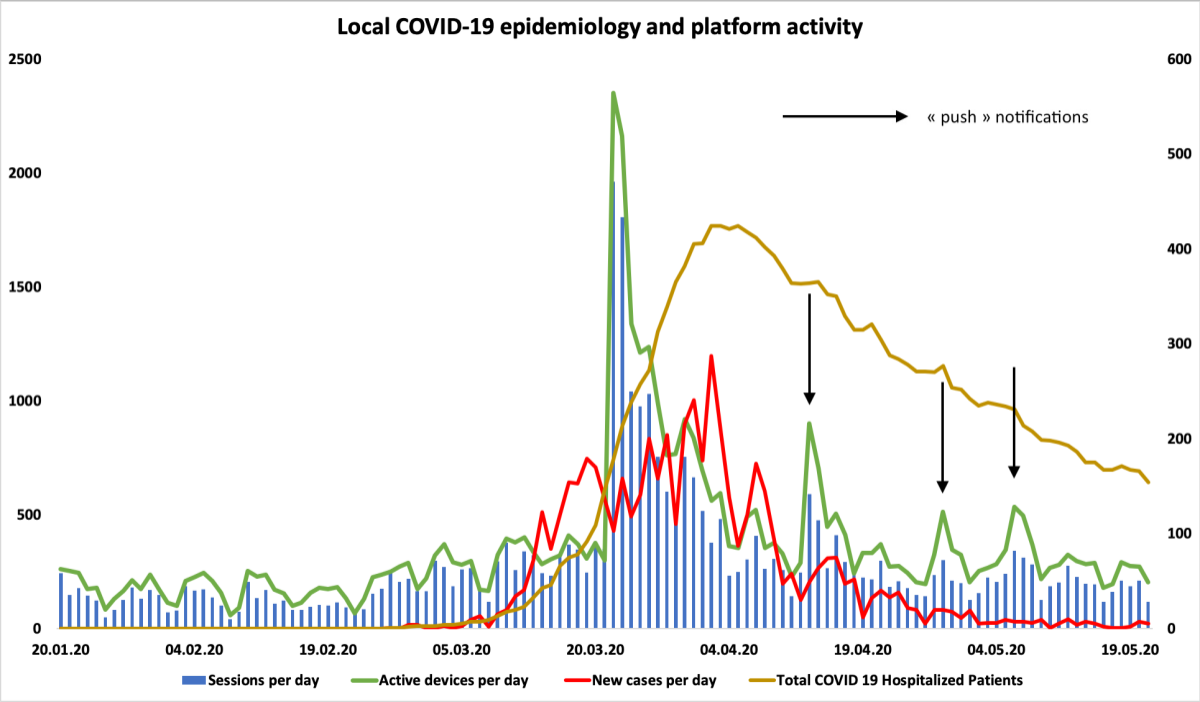
User activity compared with local coronavirus disease (COVID-19) epidemiological data.

Figure 2 shows mHealth platform use compared with COVID-19 local epidemiological data. Maximum platform activity was observed the day following targeted dissemination, with an increased use that paralleled the increase in newly diagnosed cases. An increase in user activity, measured by active devices and sessions per day, was observed when push notifications were sent to inform users of new or updated content related to COVID-19.

## Discussion

### Principal Findings

As part of the response plan to the COVID-19 crisis, our study focused on disseminating locally endorsed and validated information for HCWs during the pandemic. A locally developed mHealth solution was adopted by institutional leadership as an information dissemination channel for COVID-19–related content, following a recent successful pilot study in the Children’s Hospital of our institution [12]. In this quantitative study, we observed a significant increase in user activity immediately after targeted dissemination. During a period of 2 weeks, 912 people downloaded the mHealth platform and user activity, measured by the number of users and sessions per day, increased fourfold, showing a significant increase from baseline activity. The increase in user activity was especially notable in the first 2 weeks following targeted dissemination, with HCWs showing a high interest in COVID-19–related content; in total, 71.2% (n=7740) of content consulted during this period was COVID-19–related, an average of 550 documents per day.

Variations in user activity between observation periods could be explained by local epidemiology and circumstances. In fact, our institution centralized all regional COVID-19–related care and paused all nonurgent surgical and outpatient activity. Thus, the main daily clinical activity in our institution during Period B was COVID-19–related. As shown in Figure 2, this period happened early in the local epidemiology, when the number of new daily cases and new hospitalized patients were highest, and seemed to correspond to the highest user activity on the platform. Therefore, we postulate that increased user activity in this period was related to the acute need of HCWs to assimilate knowledge concerning COVID-19–related care. Subsequently, the standardized aspect of COVID-19–related care combined with the lack of other types of medical activity and the decrease in new cases and hospitalizations, as well as the experience and knowledge gained by HCWs during previous weeks could explain the decrease in user activity in Period C. Interestingly, during Period C, user activity stayed significantly higher than before targeted dissemination of the platform (Period A). This relative increase could be explained by the increased number of total users and might show that HCWs adopted the platform as a knowledge source. The latter might be confirmed by the fact that the percentage of COVID-19–related documents viewed decreased during this period, amounting to only 29.8% of total content viewed, while the number of hospitalized patients remained stable. This might suggest that HCWs had a general interest in the content offered on the platform.

The COVID-19 pandemic presents several unprecedented challenges to international and local governments as well as to health authorities and institutions. Prevention of infection, reduction of the transmission rate, and adequate management of patients with COVID-19 in the context of a constantly evolving body of evidence are currently a priority for jurisdictions worldwide. An infodemic has been occurring in parallel with the pandemic, as unfiltered information is easily accessible, which may allow fake news and false rumors to spread. In its last situation report, the WHO warned about fake products used for diagnosis, prevention, and treatment of COVID-19, showing an urgent need for better awareness among the general public, as well as HCWs [21].

Hospital reorganization and restructuring created a reality where a large number of HCWs from different clinical backgrounds were required to learn a large amount of new clinical procedures and practices in a short period of time. In addition, medical students worldwide have played an active role in the response to this crisis [22], and represent a special population of HCWs that have not completed their training, and thus might require more supervision and could benefit from easily accessible validated information [12].

A solid communication strategy requires an assessment of its efficiency, which can be difficult to achieve since this requires feedback from the sender on how the dissemination process was perceived and from the targeted audience on whether and how the information was received. A high discrepancy rate was reported during the H1N1 crisis, with 81% of local health departments perceiving their ability to disseminate information as very good or excellent, while only 52% of surveyed physicians reported receiving information, and as few as 16% reported using this information [11]. A systematic review showed that same discrepancy and showed that the effectiveness of communication between health authorities or leadership and HCWs is not well documented in the literature and needs to be evaluated in a more rigorous manner [10]. The same team found that email was a preferred communication method compared to fax, SMS text messaging, or no message at all, but did not investigate an mHealth solution at that point [12].

mHealth solutions are increasingly used, gaining relevance among health professionals, and may represent an interesting solution to disseminate information to and among HCWs [13-15]. Other technological initiatives have been described during the COVID-19 pandemic, such as the elaboration of an electronic health record tool to support clinical care for patients with COVID-19 and to monitor different clinical characteristics (case identification, isolation procedures, adherence to patient screening, real-time data sharing) in their hospital [23]. This type of initiative underlines the role of evolving technology, especially when it concerns time-sensitive information, and emphasizes the need for quick reactions from medical leadership and information technology staff.

The role of our mHealth platform has been previously described for students’ clinical training and exam preparation as well as for medical residents’ training [16]. In a recent quantitative and qualitative analysis involving HCWs of the Children’s Hospital in HUG completed at the beginning of the COVID-19 epidemic in Switzerland, the platform appeared to be of value and it was rapidly implemented and seen as time-effective and informative [12]. In the current study, we confirm that the platform was effectively used during Period A, when 31.2% of documents were COVID-19–related. We also showed that an mHealth solution could offer stakeholders quantitative feedback on user activity, which might be a valid and appropriate method for measuring the reach of information. Indeed, in this study, 2 weeks after the large-scale drafting and dissemination of local guidance, user activity and content use increased significantly. This increased user activity and the high percentage of COVID-19–related content use may be further evidence of the pertinence of mHealth solutions as efficient communication tools to increase the reach of validated knowledge to HCWs.

Push notifications are another interesting feature of mHealth solutions, and could have the potential to increase the reach of information as well as knowledge updates for HCWs. In our institution, as illustrated in Figure 2, user activity increased when push notifications concerning updates and new content were sent. This is a major advantage of mHealth solutions compared to classic communications methods, as it might reduce the need for HCWs to actively seek new and updated information and increase the visibility of knowledge updates.

Another potentially interesting aspect of our platform is that many of our medical students were already in possession of the mobile app and familiar with accessing local recommendations, which might have helped with their integration into clinical practice.

Finally, thanks to increased content visibility, a larger amount of medical content was made available through the platform and more medical and paramedical specialized divisions in our institution requested to share their own COVID-19–related content about specific and specialized clinical situations.

The main limitations of this study are the lack of assessment of adherence to the drafted guidance and the lack of qualitative user feedback, both of which could be considered important markers of successful reach. A qualitative assessment of the platform was recently performed in the Children’s Hospital of the HUG, which showed that HCWs felt reassured by content dissemination through the platform, found it time-efficient, and had less need to seek other information sources [12]; however, an assessment of adherence to the guidance needs to be included in future research. Nevertheless, we believe that sustained user activity, a high percentage of COVID-19–related content use, and increased activity observed shortly after push notifications suggest that disseminated information properly reached medical staff. Moreover, we believe that further validation of knowledge reach could be achieved with short, mandatory, and automatically generated quizzes concerning specific content items, potentially providing stakeholders with a higher degree of feedback and quantitative measurement of content reach.

### Conclusions

An mHealth solution seemed to be an effective and quick way to increase the reach of validated information to medical staff, in particular for time-sensitive and rapidly evolving medical guidance. Push notifications are an interesting feature of mHealth solutions for knowledge updates and could further increase the reach of information. In addition, real-time statistics could give quantitative feedback on the efficiency of the information dissemination strategy. Further research should be done to assess the clinical impact of mHealth solutions, such as adherence to validated guidance, quality of clinical practice, and patient outcomes. The use of automatically generated and mandatory quizzes concerning important content could provide stakeholders with additional and valuable feedback on content reach and would be the subject of future research. Finally, a medicoeconomical assessment should be done to fully understand the impact of mHealth solutions on information dissemination within institutions.

## Abbreviations

COVID-19: coronavirus disease
HCW: health care worker
HUG: Geneva University Hospitals
mHealth: mobile health
SARS-CoV-2: severe acute respiratory syndrome coronavirus 2
WHO: World Health Organization
